# Neoadjuvant chemoradiotherapy followed by resection for esophageal cancer: clinical outcomes with the ‘CROSS-regimen’ in daily practice

**DOI:** 10.1093/dote/doab068

**Published:** 2021-09-23

**Authors:** Marissa Cloos-v.Balen, Edmée S H Portier, Marta Fiocco, Henk H Hartgrink, Alexandra M J Langers, Karen J Neelis, Irene M Lips, Femke P Peters, Marije Slingerland

**Affiliations:** Department of Medical Oncology, Leiden University Medical Center and Groene Hart Ziekenhuis Gouda, Gouda, the Netherlands; Department of Radiation Oncology, Leiden University Medical Center, Leiden, the Netherlands; Department of Biomedical Science, Medical Statistical Section, Leiden University Medical Center, Leiden, the Netherlands; Department of Surgery, Leiden University Medical Center, Leiden, the Netherlands; Department of Gastroenterology and Hepatology, Leiden University Medical Center, Leiden, the Netherlands; Department of Radiation Oncology, Leiden University Medical Center, Leiden, the Netherlands; Department of Radiation Oncology, Leiden University Medical Center, Leiden, the Netherlands; Department of Radiation Oncology, Leiden University Medical Center and The Netherlands Cancer Institute, Leiden, the Netherlands; Department of Medical Oncology, Leiden University Medical Center, Leiden, the Netherlands

**Keywords:** adverse effects, chemoradiotherapy, esophageal neoplasms, esophagectomy, neoadjuvant therapy

## Abstract

**Background and objectives:**

Since the first results of the Dutch randomized CROSS-trial, neoadjuvant chemoradiotherapy (CRT) using carboplatin and paclitaxel followed by resection for primary resectable nonmetastatic esophageal cancer (EC) has been implemented as standard curative treatment in the Netherlands. The purpose of this retrospective study is to evaluate the clinical outcomes of this treatment in daily practice in a large academic hospital.

**Methods:**

Medical records of patients treated for primary resectable nonmetastatic EC between May 2010 and December 2015 at our institution were reviewed. Treatment consisted of five weekly courses of carboplatin (area under the curve 2) and paclitaxel (50 mg/m^2^) with concurrent external beam radiotherapy (23 fractions of 1.8 Gy), followed by transthoracic or transhiatal resection. Data on survival, progression, acute and late toxicity were recorded.

**Results:**

A total of 145 patients were included. Median follow-up was 43 months. Median overall survival (OS) and progression-free survival (PFS) were 35 (95% confidence interval [CI] 29.8–40.2) and 30 (95% CI 19.7–40.3) months, respectively, with corresponding 3-year OS and PFS of 49.6% (95% CI 40.4–58.8) and 45.6% (95% CI 36.6–54.6). Acute toxicity grade ≥3 was observed in 25.5% of patients. Late adverse events grade ≥3 were seen in 24.8%, mostly esophageal stenosis.

**Conclusion:**

Neoadjuvant CRT followed by resection for primary resectable nonmetastatic EC in daily practice results in a 3-year OS of 49.6% (95% CI 40.4–58.8) and PFS of 45.6% (95% CI 36.6–54.6), compared with 58% (51–65%) and 51% (43–58%) within the CROSS-trial. The slightly poorer survival in our daily practice group might be due to the presence of less favorable patient and tumor characteristics in daily practice, as is to be expected in daily practice. Toxicity was comparable with that in the CROSS-trial and considered acceptable.

## INTRODUCTION

Esophageal cancer is the eight most common cancer in the world, with nearly 500,000 new cases each year.^1^ Surgical resection has long been the standard treatment for nonmetastatic esophageal cancer, but despite improvements in preoperative staging, patient selection, surgical techniques and postoperative care, the prospect for cure remained unsatisfactory.^2^ Multiple studies have focused on improving outcomes through the addition of chemotherapy, radiotherapy or both in a neoadjuvant or adjuvant setting. Meta-analyses by Sjoquist *et al*. from 2011,^3^ comparing neoadjuvant chemotherapy or chemoradiotherapy (CRT) followed by resection to surgery alone, showed a survival benefit for both neoadjuvant chemotherapy and CRT.^3^ In our institution, we introduced neoadjuvant CRT followed by resection for primary resectable nonmetastatic esophageal cancer as the standard curative treatment in response to publication of the first results of the Dutch randomized ‘CROSS-trial’ in 2010.^4^ The long-term results of this trial^5^ showed a significant overall survival (OS) and progression-free survival (PFS) benefit and significantly improved locoregional disease control with neoadjuvant CRT followed by resection compared with resection alone. The postoperative complication rates were comparable and grade ≥3 toxicity was infrequently seen.

Clinical outcomes in daily practice, however, are usually worse than results obtained within trials. It is important to have insight in clinical outcomes in daily practice, for both patients and treating physicians.

The aim of this study was to determine clinical outcomes (OS and PFS) and toxicity in patients treated with neoadjuvant CRT followed by resection in daily practice.

## MATERIALS AND METHODS

### Patient selection

All consecutive patients with primary resectable esophageal cancer scheduled for neoadjuvant CRT according to the CROSS-regime followed by resection between May 2010 and December 2015 were included. Neuroendocrine histology, treatment for recurrent esophageal cancer and a 6-week CRT scheme followed by resection were exclusion criteria.

### Data collection

With approval of the Medical Ethical Committee, characteristics of patients, tumors, treatment, experienced adverse events and outcomes, including definitive pathology report with pathological response according to Mandard^6^, were retrieved from the medical records. Recorded pretreatment characteristics were age, sex, World Health Organization (WHO) performance score, comorbidity according to Adult Comorbidity Evaluation—27, weight loss, tube feeding, dysphagia, stage of disease according to the 7th edition of the TNM Classification of Malignant Tumors, tumor location (proximal, middle, distal), histological type, tumor length measured during endoscopy (if not available, measured on computed tomography-scan [CT-scan]) and largest tumor diameter on axial CT-scan. Dysphagia scores were generally not clearly defined, and therefore interpreted from patients’ charts according to Knyrim *et al*.^7^

Acute toxicity as a result of CRT was scored according to the Common Terminology Criteria for Adverse Events v4.0. Acute adverse events were defined as those that occurred during or within 6 weeks after the end of treatment (about the time of the surgery), and late toxicity as those that occurred beyond 6 weeks after CRT. Surgical complications were scored according to the Clavien Dindo^8^ classification of surgical complications. Late complications of the surgery were defined as those which occurred >90 days after surgery. Thirty-day mortality after surgery was recorded.

### Diagnostic process

Diagnostic work-up included an esophagogastroduodenoscopy with biopsy for histological confirmation. All patients underwent a CT-scan or positron emission tomography scan. When on CT scan a T4 component was suspected, an endoscopic ultrasound was performed. An ultrasound of the neck was made when cervical lymph nodes were not adequately depicted on scans or involvement of the cervical lymph nodes was suspected. All patients were discussed in a multidisciplinary team of at least a surgeon, gastroenterologist, medical oncologist, radiation oncologist, (nuclear) radiologist and pathologist.

### Treatment

All patients underwent external beam radiotherapy using 6-MV beams. A total dose of 41.4 Gy was given in 23 fractions of 1.8 Gy, five fractions per week. The gross tumor volume (GTV) consisted of the primary tumor in de esophagus with pathological lymph nodes contoured separately (GTV-node). To obtain the clinical target volume (CTV), the GTV-tumor was expanded along the esophagus in cranial and caudal direction with 3 and 1 cm in other directions. If necessary, the CTV was expanded beyond 1 cm to include the entire periesophageal fatty tissue. When the caudal extension of the tumor reached into the stomach, the caudal margin was decreased to 2 cm. GTV-node was expanded with a margin of 0.5 cm in all directions. The CTV was adjusted to anatomical boundaries, e.g. the vertebrae, heart and lungs. The planning target volume included the CTV with a 1 cm margin. CT-based three-dimensional conformal treatment planning was used. Maximum accepted dose in the spinal cord was 50 Gy and maximum mean lung dose was 16 Gy.

Concurrent chemotherapy consisted of five weekly administrations of carboplatin (area under the curve = 2) and paclitaxel (50 mg/m^2^), preferably starting at the day of the first radiotherapy fraction.

A new CT-scan was performed 2–3 weeks after completion of neoadjuvant CRT. If distant metastases (DMs) were absent, the resection was performed ~6 weeks after completion of CRT. A transthoracic or transhiatal approach was used, depending on the location of the tumor; for tumors located proximal of the distal one-third, a transthoracic resection with a two-field lymph node dissection was performed. A transhiatal resection with two-field lymph node dissection was performed for tumors in the distal one-third of the esophagus and for tumors involving the esophagogastric junction, including resection of nodes along the hepatic artery, splenic artery and left gastric artery.

### Follow-up

Follow-up was performed at the surgery department. Outpatient visits were scheduled every 3 months during the first year after treatment. From the second year, follow-up was performed every 6 months until 5 years after treatment. Recurrences were registered at the date of first diagnosis of recurrence. When a patient was lost to follow-up, the Municipal Personal Records Database was consulted for data about survival. We followed patients ultimately till 29 August 2016.

### Statistical analysis

Descriptive statistics were used to provide information about baseline characteristics. For normally distributed data, a mean (standard deviation) and median were estimated. For data with a skewed distribution, the median and range were provided.

Survival was estimated from start of treatment to date of death, with censoring at date of last follow-up contact for patients still alive. PFS was defined as the interval between start of treatment and the occurrence of disease progression resulting in primary (or perioperative) irresectability of disease, locoregional recurrence (after completion of therapy), DMs (during or after completion of treatment), or death from any cause. To estimate the cumulative incidence of locoregional recurrence and DM, a competing risk model^9^ with death as competing event was employed. Kaplan–Meier’s methodology was use to estimate survival outcomes. An intention to treat analysis was performed, including all patients that started treatment. To study the effect of response to the therapy defined at surgery, a Cox proportional hazard regression model was estimated. Analysis was performed with SPSS 23.0 (IBM). All analysis concerning the competing risk model was performed with the mstate^10^ library in R software environment.

## RESULTS

### Population

Between May 2010 and December 2015, 145 patients started treatment with neoadjuvant CRT followed by surgery. Baseline characteristics are shown in Table 1. Mean age was 64 years. The majority of the patients presented with a cT2 (15.2%) or cT3 (76.6%) tumor. A total of 33 patients (22.8%) had a clinical N0 stage at diagnosis, 71 patients N1 (49%) and 31 patients N2 (25.5%). In all, 78% of patients had a good performance score: 59.3% had a WHO performance status of 0 and 16.6% 1. Histology showed a squamous cell carcinoma in 32 patients (22.1%) and adenocarcinoma in 133 patients (77.9%).

**Table 1:** Baseline patient characteristics

Characteristics		*N*	(%)
Age	Median (range)	64	(25–82)
Sex			
Male		114	(78.6)
Female		31	(21.4)
WHO performance score			
WHO 0		86	(59.3)
WHO 1		24	(16.6)
WHO 2		3	(2.1)
Unknown		32	(22.1)
ACE 27 score			
None		42	(29)
Mild		72	(49.7)
Moderate		21	(14.5)
Severe		10	(6.9)
Tumor histology			
Adenocarcinoma		113	(77.9)
Squamous cell carcinoma		32	(22.1)
Tumor location			
Middle		19	(13.1)
Low		112	(77.2)
GEJ		14	(9.7)
Clinical tumor stage (TNM 7)			
cT2		22	(15.2)
cT3		111	(76.6)
cTx		12	(8.3)
Clinical nodal stage			
cN0		33	(22.8)
cN1		71	(49)
cN2		37	(25.5)
cN3		1	(0.7)
cN+ (not specified)		1	(0.7)
cNx		2	(1.4)

ACE, Adult Comorbidity Evaluation; WHO, World Health Organization; GEJ, Gastro Intestinal Junction.

### Treatment

All patients completed radiotherapy as intended. A total of 126 patients (86.9%) completed the full five cycles of chemotherapy, 16 patients (11%) completed four cycles and three patients (2.1%) completed three cycles. Patients who did not complete all five cycles showed a worse survival, with a median OS of only 18 versus 39 months if all five cycles were completed. Hematological toxicity was the main reason for discontinuation. A total of 130 patients underwent resection (89.7%). Reasons for not undergoing resection were preoperative metastatic disease in eight patients (5.5%) or irresectable tumor during the procedure in seven patients (4.8%).

### Outcome

**Fig. 1:**
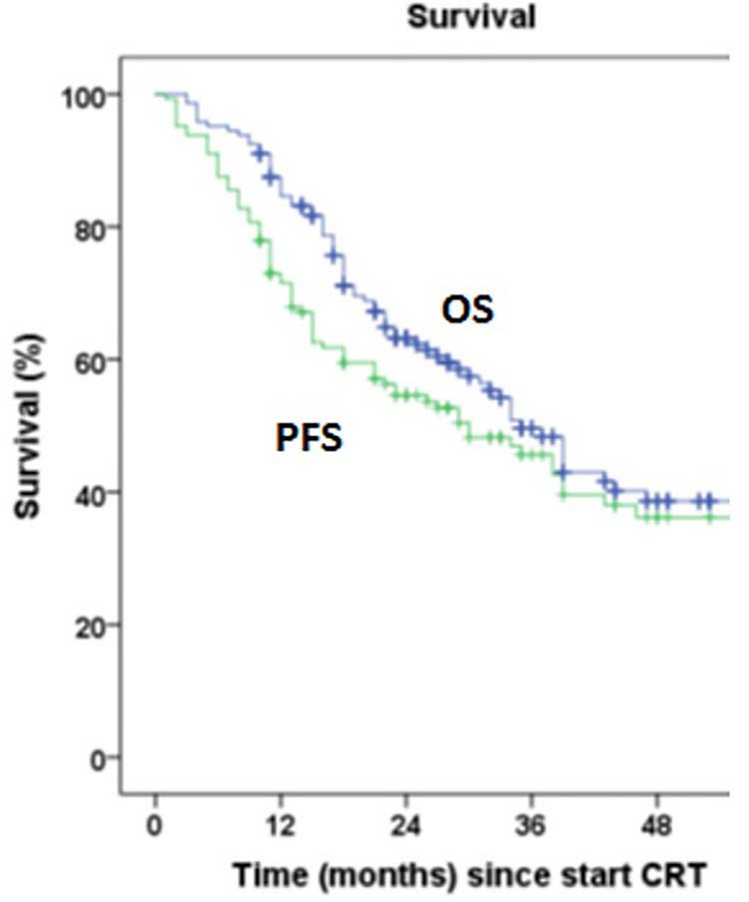
Overall survival and progression-free survival (in months) since start of chemoradiotherapy. CRT, chemoradiotherapy; OS, overall survival; PFS, progression-free survival.

**Fig. 2:**
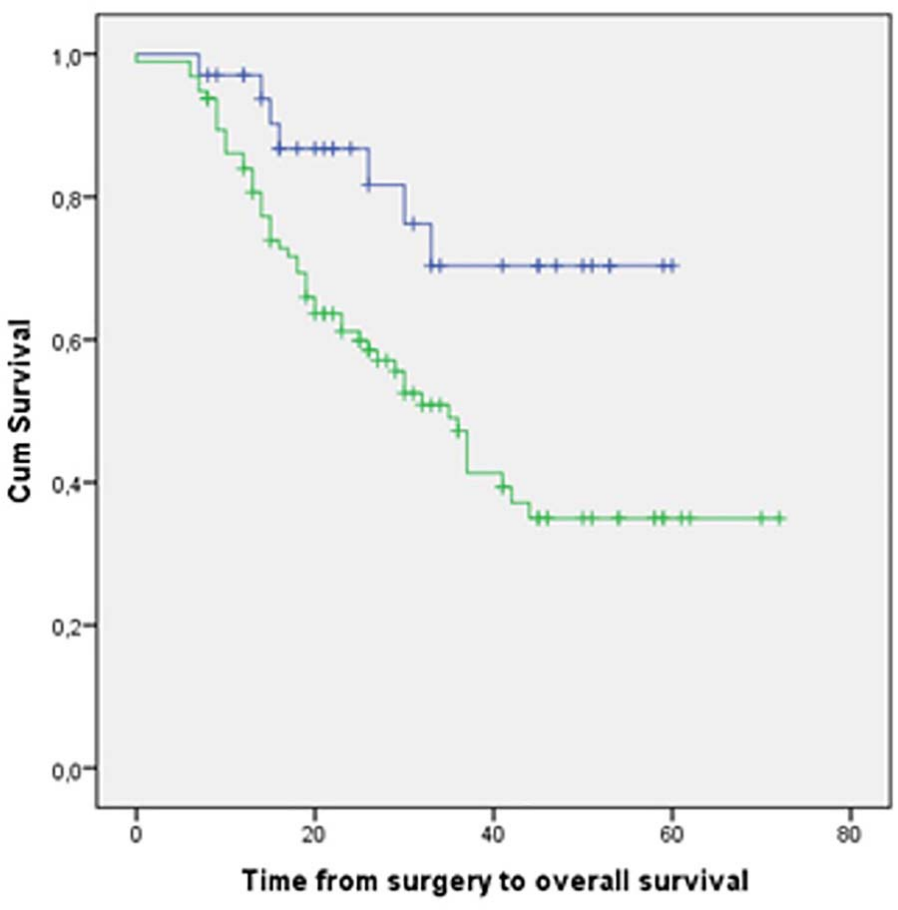
Overall survival comparing complete pathological response with no complete pathological response. Blue line (**_**) indicates complete pathological response; green line (**_**) indicates no complete pathological response.

**Fig. 3:**
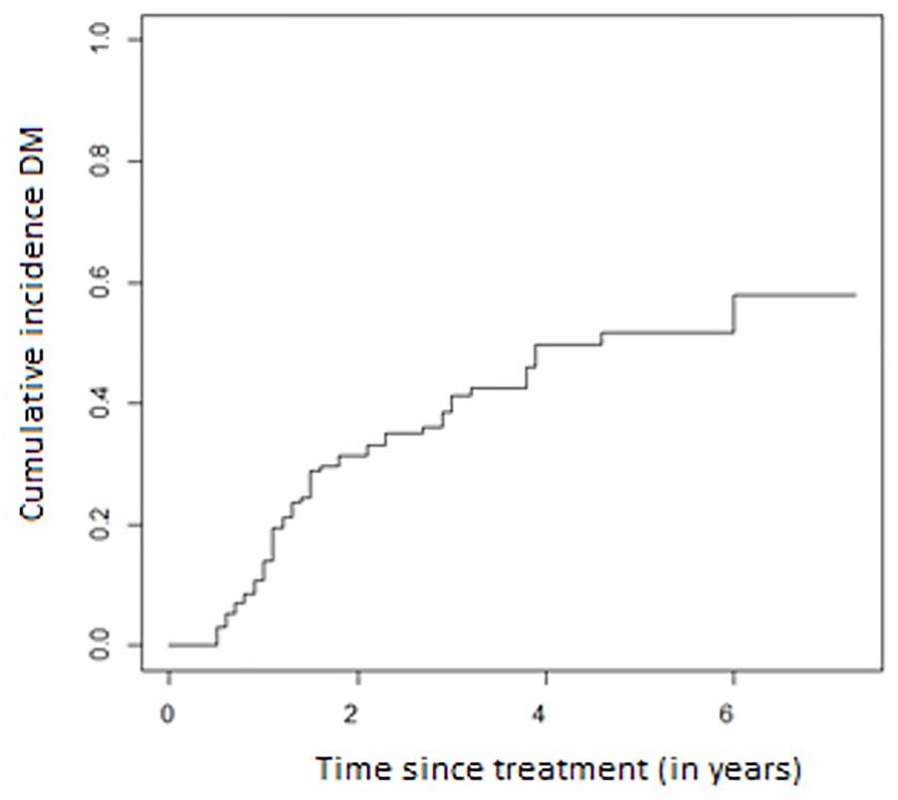
Cumulative incidence of distant metastasis.

**Fig. 4:**
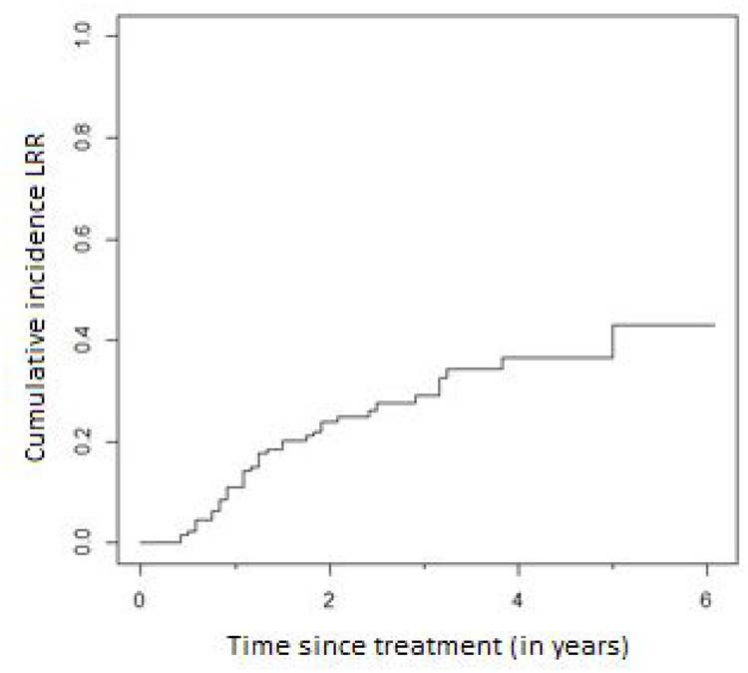
Cumulative incidence of locoregional recurrence.

Median follow-up was 43 months (95% confidence interval [CI] 32.5–53.4 months). Median OS was 35 months (95% CI 29.7–40.2 months). Three-year OS and PFS were 49.6% (95% CI 40.4–58.8) and 45.6% (95% CI 36.6–54.6), respectively (Fig. 1). A complete pathologic response (based on the conclusion of the pathologist) was found in 30 patients (23.1%) and a partial response in 95 (73.1%). Patients who did not experience a complete response had a worse OS with a hazard ratio equal to 2.8 (95% CI 1.20–6.54) compared with the group having a complete pathologic response (Fig. 2). During follow-up, 31.01% (22.89–39.13) of patients who started CRT developed local regional recurrence (LRR) and 45.33% (36.48–54.19) developed DM. Most LRR and DM developed in the first 3 years after treatment (Figs 3, 4).

### Toxicity

In total, 37 patients (25.5%) experienced an adverse event of grade ≥3 during CRT or in the 6 weeks after completion. Toxicity was predominantly gastrointestinal (esophagitis) and asymptomatic hematological (neutropenia and thrombocytopenia). Acute postoperative grade ≥3 events occurred in nine (6.5%) patients who underwent surgery. These complications were paresis of the nervus recurrens in two patients (1.5%), anastomotic leakage in two patients (1.5%), wound infection in four patients (3%) and leakage of the jejunal fistula in one patient (0.8%).

Late postoperative grade ≥3 events occurred in 34 patients (24.8%). The most frequent side-effect was stenosis of the esophagus in 26 patients (20%). Other side-effects were stenosis of the pylorus and ileus.

## DISCUSSION AND CONCLUSION

In this cohort of 145 patients, treatment with neoadjuvant CRT and surgery led to a 3-year OS of 49.6% (95% CI 40.4–58.8) and a 3-year PFS of 45.6% (95% CI 36.6–54.6), compared with 58 and 51% within the original CROSS-trial.^5^

Looking at the patient characteristics in both groups (our cohort and the patients reported in the CROSS-trial), there are some clear differences. First, the patients in our cohort scored worse on the WHO performance score in comparison with the ‘CROSS’ cohort: 59.3% had a WHO performance scale (PS) of 0 compared with 81% in the CROSS-trial. Second, the median age in our cohort is 4 years higher: 60 versus 64 years old. We know from previous studies that older age and functional impairment are associated with a higher operative mortality and poor 5-year survival.^11–14^

Third, our cohort includes patients with both cN1 and cN2 disease (25.5%), whereas in the CROSS-trial, only patients with N1 nodes according to the TNM 6th edition were included. Rizk *et al*.^15^ and Talsma *et al*.^16^ showed that patient with more involved lymph nodes have a worse prognosis than the N0 and N1 staged patients. We do not know how much patients in the original ‘CROSS’ cohort would have classified as an N2 in the TNM 7th edition. There is also the possibility that we have included patients as N2, who would have classified as M+ in the TNM 6th edition. These differences might even ask for other treatment options, which could be a question in a new trial (Should we treat patients with N2, N3 disease with a more intensive chemotherapy regimen?).

Fourth, the median tumor length in our cohort was 5 cm with an interquartile range of 4–7 cm, whereas in the CROSS-trial, this was 4 cm with an interquartile range of 3–6 cm, indicating that in daily practice larger tumors are being treated. The differences in patient characteristics might explain the differences in outcome between the original CROSS-trial and our ‘real world’ data.

When we look at the toxicity and consider that our population had a worse performance score, the toxicity in our cohort is acceptable. We reported 6.5% more grade 3 hematological toxicity than Shapiro *et al*., but no treatment-related deaths. A likely explanation for the higher rate of hematological toxicity is the older age of our cohort. We had only 6.5% grade ≥3 acute postoperative complications (paresis of the nervus recurrens, anastomic leakage, wound infection or leakage of the jejunal fistula), which is less than reported by van Hagen *et al*.^4^, who reported 3% mediastinitis and 22% anastomotic leakage. This might be explained by differences in surgical techniques (e.g. *mainly* transhiatal vs. transthoracic) between centers, progressive experience of the surgeons and improvements in postoperative care.

In conclusion, our data show that in a nonselected patient cohort, the CROSS-regime is well tolerated and leads to slightly worse outcomes compared with the orginal CROSS-trial. This difference is most likely attributable to the fact that the patients in the ‘real life’ cohort have more unfavorable patient and tumor characteristics compared with the patients included in the original trial.

## Conflict of interest

The authors declare that they have no conflict of interest.
